# Non-Contact Spirometry Using a Mobile Thermal Camera and AI Regression

**DOI:** 10.3390/s21227574

**Published:** 2021-11-15

**Authors:** Luay Fraiwan, Natheer Khasawneh, Khaldon Lweesy, Mennatalla Elbalki, Amna Almarzooqi, Nada Abu Hamra

**Affiliations:** 1 Department of Electrical, Computer and Biomedical Engineering, Abu Dhabi University, Abu Dhabi 55991, United Arab Emirates; 1065023@students.adu.ac.ae (M.E.); 1065804@students.adu.ac.ae (A.A.); 1064647@students.adu.ac.ae (N.A.H.); 2 Department of Biomedical Engineering, Jordan University of Science and Technology, Irbid 2210, Jordan; klweesy@just.edu.jo; 3 Department of Software Engineering, Jordan University of Science and Technology, Irbid 2210, Jordan; knatheer@just.edu.jo

**Keywords:** thermal camera, non-contact spirometry, artificial intelligence regression, respiration signal, respiration rate mobile application

## Abstract

Non-contact physiological measurements have been under investigation for many years, and among these measurements is non-contact spirometry, which could provide acute and chronic pulmonary disease monitoring and diagnosis. This work presents a feasibility study for non-contact spirometry measurements using a mobile thermal imaging system. Thermal images were acquired from 19 subjects for measuring the respiration rate and the volume of inhaled and exhaled air. A mobile application was built to measure the respiration rate and export the respiration signal to a personal computer. The mobile application acquired thermal video images at a rate of nine frames/second and the OpenCV library was used for localization of the area of interest (nose and mouth). Artificial intelligence regressors were used to predict the inhalation and exhalation air volume. Several regressors were tested and four of them showed excellent performance: random forest, adaptive boosting, gradient boosting, and decision trees. The latter showed the best regression results, with an R-square value of 0.9998 and a mean square error of 0.0023. The results of this study showed that non-contact spirometry based on a thermal imaging system is feasible and provides all the basic measurements that the conventional spirometers support.

## 1. Introduction

Pulmonary diseases such as asthma, chronic obstructive pulmonary disease (COPD), pneumonia, bronchitis, pleural effusion, and lung fibrosis affect tens of millions of people all over the world [1]. The monitoring of respiratory function is of great importance in diagnosing these diseases. One of the most widely used methods of diagnosing and assessing the progression of respiratory diseases is spirometry. In conventional spirometry, the patient wears a mouthpiece for breathing and puts on a nose clip. The patient is instructed to breathe in a certain procedure so that several parameters can be measured during the breathing protocol. The patient is instructed to inhale and exhale into the spirometer mouthpiece at a certain breathing rate and force, then the spirometer calculates the breathing rate and the volumes of inhaled and exhaled air during the measurement procedure. As an example of using the spirometer in clinical procedures to diagnose obstructed airways, the ratio of the forced expiratory volume in one second (FEV1) to the forced vital capacity (FVC) (i.e., FEV1/FVC) is measured, where FEV1 represents the maximum amount of air the subject can exhale in 1 s and FVC represents the greatest volume of air the subject can exhale after a deep inhale [2]. A low FEV1/FVC value means that the amount of air that the airways can quickly exhale out of the lungs is reduced, which indicates that the airways are obstructed [3,4], while an increase in the FEV1/FVC value results in a restrictive condition that affects the ability to inhale. According to the American thoracic society, an FEV1/FVC value of 0.7 or above is considered normal for adults, while a value of 0.85 or above is considered normal for children aged 5 to 18 years [5].

The current techniques that are used for spirometry can be classified into two categories: contact spirometry and non-contact spirometry. Contact spirometry is the traditional and the most common method in clinical practice. This conventional way of performing spirometry can be uncomfortable for patients, since it requires them to keep the spirometer in their mouth for up to 30 min. Moreover, after each patient use, the spirometry mouthpiece is subjected to a sterilization procedure to prevent any cross-infection among the patients. There have been several techniques developed for non-contact spirometry. Most of these techniques depend on body movement detection [6,7], photoplethysmography (PPG) [8], and imaging techniques. Droitcour et al. [6] and Mostov et al. [9] used Doppler radar to measure the movement of the body due to heart and respiration signals. For this, they used a high-quality CMOS Doppler radar sensor and verified their results against standard heart and respiration devices. An image-based system was used by Lin et al. [10], where they developed a method based on harmonic analysis of body motion to detect changes in the respiration rate pattern. The second category of non-contact spirometry techniques is based on signal analysis of the PPG signals. In this category, the change in blood volume in the blood vessels is measured by observing changes in light absorption. The measured PPG signal is normally modulated by the respiratory signal (activity) due to movement of the chest cavity during blood flow. These techniques that are based on the analysis of PPG signals are normally based on signal processing techniques and can provide only frequency information about respiration without any information about the changes in the lung volume. Madhav et al. [8] used a modified method of principal component analysis (PCA) to separate the heart activity from the respiration activity. The recorded PPG signal was acquired by a pulse oximeter. Another study depending on ambient light for the recording of the PPG signal was performed by Verkruysse et al. [11]. They used harmonic analysis for extraction of the different components of the PPG signal including the respiration activity. Al-Naji et al. [12] used an ultrasonic PING sensor and PIC18F45 microcontroller to monitor the normal and abnormal breathing activity of the subjects continuously and instantaneously at different distances. The third category of non-contact spirometry techniques is based on thermal imaging, where measurement of the respiration activity is performed through monitoring the changes in the air temperature during inhalation and exhalation [13,14]. Murthy and Pavlidias used a highly sensitive imaging system in their measurements [14]. They used a statistical model of the recorded image to extract respiratory breathing information. Another study by Murphy et al. [15] evaluated the airflow rate using a thermal imaging system.

Most of the non-contact techniques mentioned in the literature do not provide complete respiratory values as the conventional method does, such as the volume of air inhaled and exhaled during the measurement protocols [6,8,9,10,14,16,17]. Moreover, most times, they require sophisticated techniques, a special setup, and special hardware, while conventional spirometry is considered simple and cheap, with the only disadvantage of requiring direct contact with the patient, which could produce cross-infection and discomfort. Therefore, any non-contact spirometry system should address these issues. This present work provides a feasibility study of using a mobile thermal camera to replace the conventional spirometer for measurements of the respiration rate and the volume of inhaled and exhaled air in and out of the lungs. The purpose of this study was to build a simple non-contact respiratory measurement system using a mobile thermal camera. The targeted measurements included the respiration rate and the volume of inhaled and exhaled air based on artificial intelligence (AI) regressors.

## 2. Materials and Methods

**Figure 1:**
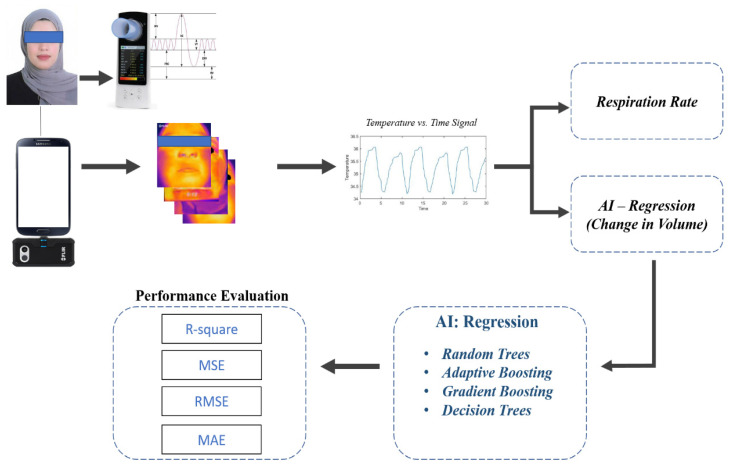
Graphical abstract of the proposed work.

The complete structure of the proposed study is shown in Figure 1. A mobile phone, with a thermal camera connected to it, was used to acquire thermal images from the face of the subject using a mobile application that was built specifically for this purpose. The mobile application extracts the respiration signal and exports it to a back-end server for further spirometry analysis of the inhaled and exhaled air volumes, which was performed based on the AI regression approach. The spirometry measurements were carried out using a conventional spirometer.

### 2.1. Thermal Image Acquisition System

The thermal image acquisition system consisted of a mobile phone (Samsung S6) with an Android platform and a FLIR I thermal camera [18]. The camera produced images with thermal resolution of 160 × 120 pixels with a pixel size of 12 microns. Its accuracy was around 3 °C or ±5% of difference between the ambient and the scene temperature. Both still and video thermal images could be recorded, with video images recorded at a rate of 9 frames per second. A special application that used the FLIR I software development kit (SDK) (available at https://developer.ir.com/mobile/ironesdk/, accessed on 10 October 2021) was built for image acquisition. Moreover, the application used the OpenCV android SDK library for image processing. The image acquisition rate was fully controlled by the FLIR I SDK, with a static value of 9 frames per second. The application detected the region of interest (the area under the nose or the mouth) initially using a regular camera image and calculated the average temperature in that region. The application also specified the duration of the recording; a default duration of 20 s was used initially. The recorded signal of temperature versus time was displayed by the application and transmitted to a PC for further spirometry measurements.

### 2.2. Respiration Rate Measurement

**Figure 2:**
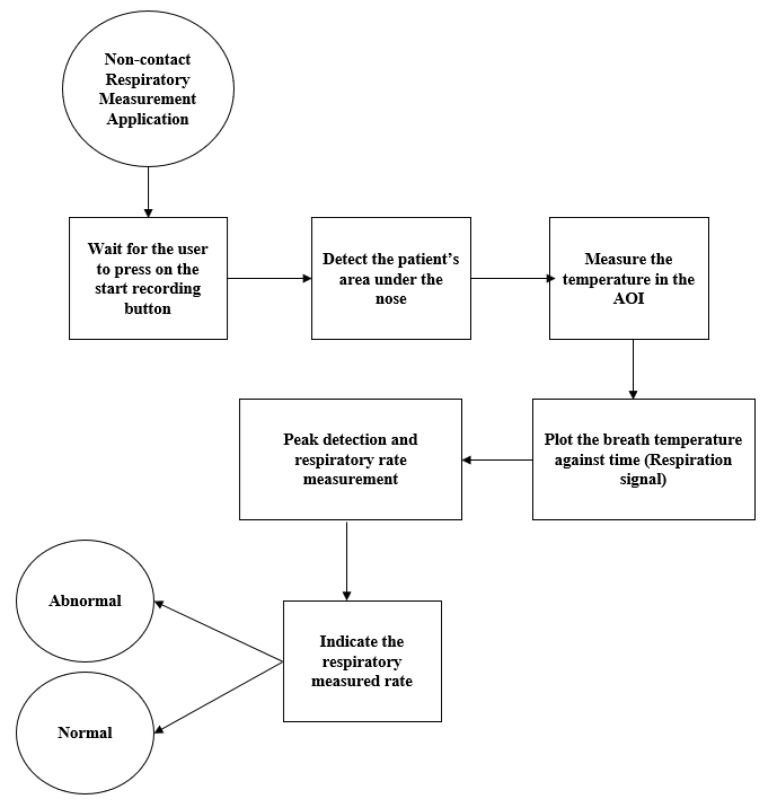
Mobile application workflow diagram.

The mobile application that was built for this study was used for two purposes: the first one was to record and transmit the respiration signal vs. time to a PC for further measurements, and the second one is was calculate the respiration rate from the recorded signal by finding the number of peaks (local maxima) in that signal divided by the duration of the recording. The mobile application indicated whether the respiration rate was normal (15–24 breaths/min) or abnormal. The flow chart of the mobile application is shown in Figure 2.

### 2.3. Spirometry Measurements

**Figure 3:**
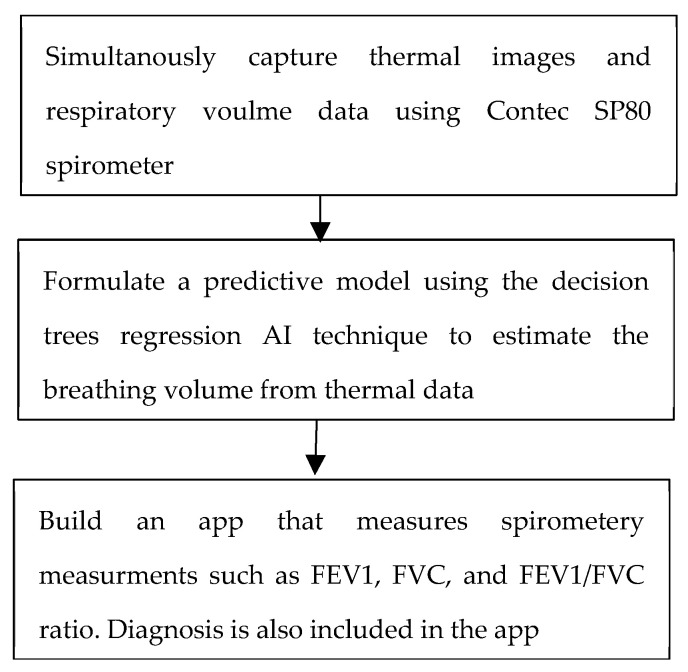
Plan of the spirometry measurement system.

**Figure 4:**
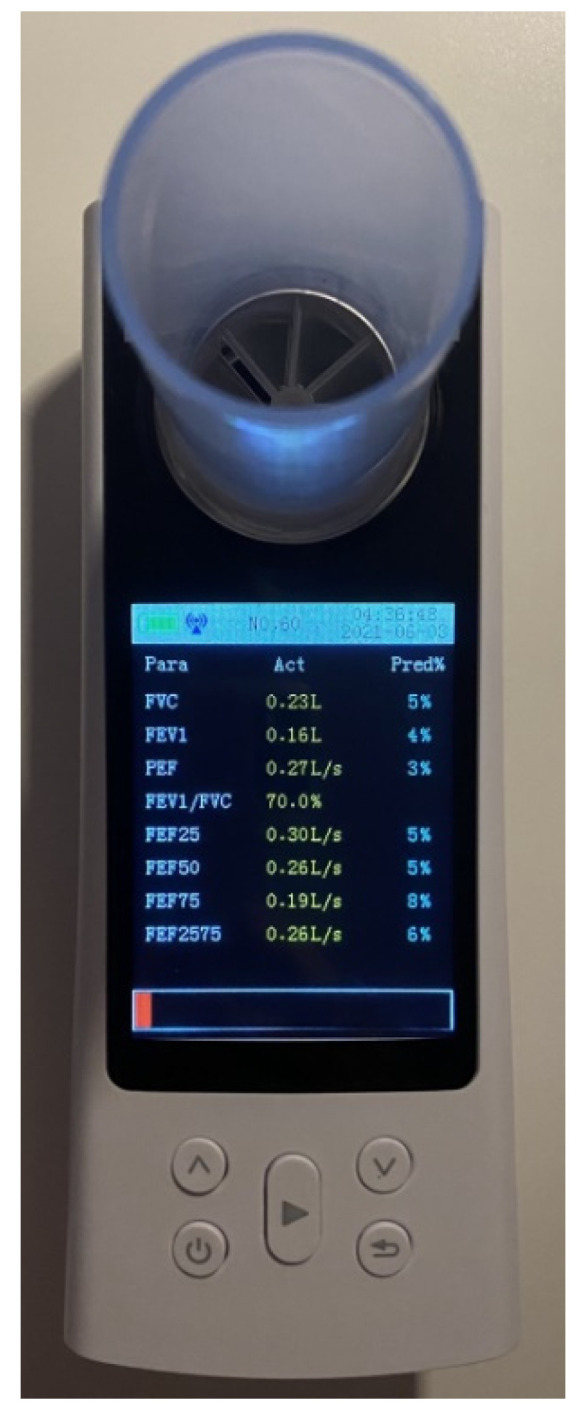
SP80B spirometer, Contec Medical Systems.

The spirometry measurement approach adopted in this work was an artificial intelligence system based on regression trees. The system’s plan of operation is shown in Figure 3. To be able to build such a system, a conventional spirometer was used to provide data about the change in lung volume during measurements. These measurements provided the ground truth for the training and testing of the AI system. The spirometer used in this study was a Contec SP80 healthcare portable medical patient spirometer (shown in Figure 4) [19]. Data were acquired from different subjects, where each time, the change in lung volume during the respiration protocol was recorded.

#### 2.3.1. Subject

The measurements used in this study were taken from 18 subjects. The age of the subjects ranged from 16 to 28 years, with an average age of 22.5. The subjects’ mass was in the range of 54 to 90 kg, with an average value of 67.5 kg, while their height was in the range of 159 to 188 cm, with an average value of 167 cm.

#### 2.3.2. Regression Models

**Figure 5:**
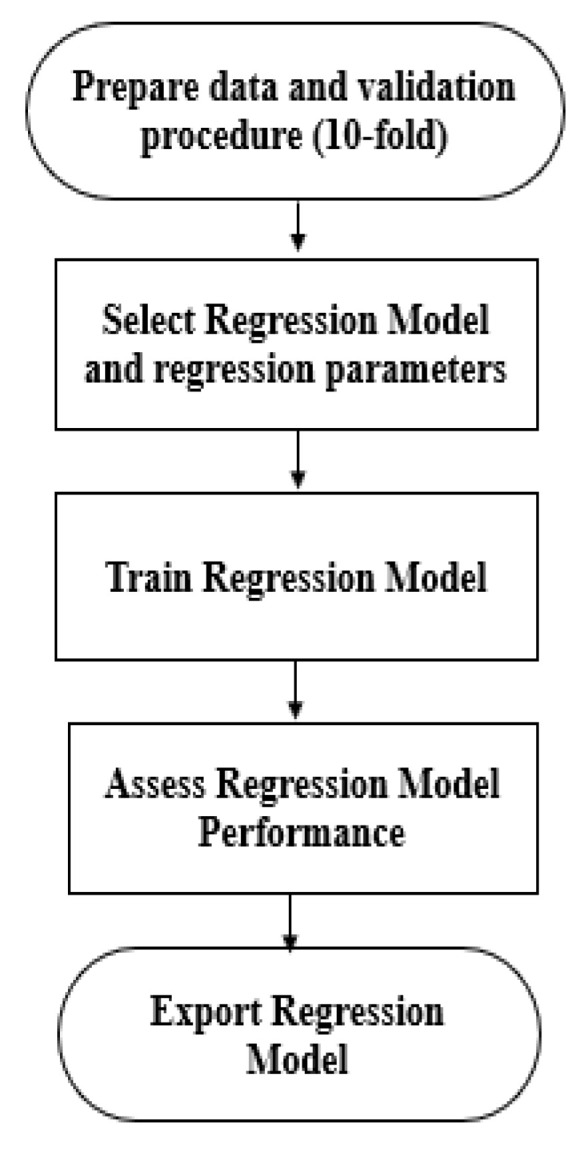
General workflow for the regression procedure.

The recorded signals of temperature versus time were used to build a regression model to predict the value of the change in volume during the respiration protocol. Several regression tree models were tested for this work. The workflow of the proposed model is shown in Figure 5.

− Decision Trees Regressor

Decision trees (DT) were first introduced by Breiman et al. in 1984 [20]. This method is widely used due to its simplicity and efficiency. The learner uses a decision tree as a predictive model for the output. The decision tree is constructed based on the recorded signal, which is considered as a feature vector (observation) for training and testing the trees. It basically splits the training dataset into smaller and smaller subsets, while simultaneously developing the corresponding decision tree in an incremental manner. The DT normally works as a top-down scheme, where each observation is used to measure the output based on a certain measure such as Gini impurity, information gain, and variance reduction. To predict a particular data point, the algorithm runs through the complete decision tree by answering “true/false” questions at every node, until arriving at the leaf node which represents the outcome. Most importantly, the same procedure is iterated multiple times to increase the accuracy of prediction. The major advantage of DT is the simplicity and the ability to handle numeric variables (regression), while the major disadvantage is the sensitivity to noise in the observations, which may cause a large variation in the decision trees and hence increase the error in the predictions [21].

− Random Forest Regressor

The random forest classifier and regressor was first introduced by Breiman in 2001 and has been subjected to several improvements since then [20,22]. The random forest is a supervised machine learning technique that deploys multiple trees running in parallel during the training stage with no interaction among the trees. The outputs of all trees are aggregated to calculate the final output of the random forest regressor. If the vector *x* contains *N* features such that *x* = [*x*_1_, *x*_2_, …, *x_N_*], these features are used to predict the output for a given input feature. The vector *X* in this study represents the thermal respiration signal recorded during the measurement protocol. The signal is used to build a predictive model to predict the value of the respiration volume. If the true measured volume is *V*, then the predicted volume is $\tilde{V}$.

This algorithm works in the following steps: firstly, it selects uncorrelated and random sub-samples from the training dataset and builds up a separate decision tree for each sub-sample. Next, to predict a particular data point, it extracts a prediction result from every decision tree. Lastly, it applies a vote procedure to all available prediction results and declares the prediction result with the greatest number of votes as the final predication. In random forests regression, the voting is the average prediction of each tree; $\tilde{V_{i}}$ is the output for of an individual tree *i*. If there are *M* trees in the random forests regressor, then the final output of the is calculated as:

$$\tilde{V}=\frac{1}{M}\overset{M}{\underset{i=1}{\sum }}\tilde{V_{i}}$$

Some of the advantages of the random forest regressor include its efficiency in handling large databases, its accuracy in performing classification and regression, and its accuracy in the case of missing data points. The main weakness of the random forest regressor is overfitting in the presence of noisy training data.

− Gradient and Adaptive Boosting Regressors

Boosting classifiers and regressors start the learning process using weak classifiers and regressors such as decision trees. They modify these weak learners to make them strong learners by trying to improve the initial model. In boosting techniques, multiple individual models are trained in a sequential way, while every successive model keeps on learning from mistakes of its predecessor model. There are several techniques for this procedure, with the most common ones being gradient boosting (GB) and adaptive boosting (AdaBoost) [21,23]. In the gradient boosting technique, several models (decision trees) are trained either in an additive or sequential manner. The learner tries to identify the weaknesses of the weak model using the gradients of the loss functions; the performance measure of the regressor used in this work was the mean square error. The different models are updated on the basis of the gradient of the loss function, and weak learners are improved. The gradient boosting regressor works as follows: firstly, it trains a decision tree with an original training dataset and extracts predictions from the trained decision tree. It then computes the residual error of the trained decision tree and stores its value as a variable *Y*. To keep on learning from previous mistakes, the next stage will utilize the calculated residual error of each stage. It repeats the same procedure for a prespecified number of trees to train the model. To predict a particular data point, it simply adds up the prediction results of all trees. In the adaptive boosting technique, the learner identifies the weak classifier (trees) using the cost function, modifies them to enhance their performance, and builds a second regression model based on these analyses. The process continues until an accurate model is completed or a certain number of models has been reached. Similar to the gradient boosting technique, firstly, it trains a simple decision tree with the original training dataset. It computes the weighted error rate (number of false predictions out of total predictions) for the decision tree. It then calculates the weight of the decision tree in the ensemble and updates the weights of all misclassified data points (wrong predictions). Lastly, it repeats the same procedure for a prespecified number of trees to train the model. To predict a particular data point, the adaptive boosting algorithm multiplies the tree’s weight by the tree’s prediction and adds up all the trees. Consequently, the tree with the highest weighting will be the most significant influencer of the final prediction.

#### 2.3.3. Performance Measures

The performance of the different regressors was evaluated based on four different measures: the coefficient of determination or R-squared (*R*^2^), the mean square error (MSE), the root means square error (RMSE), and the mean absolute error (MAE). These performance measures were calculated during the regression procedure based on 10-fold cross-validation.

The R-squared value is a statistical measure of how close the predicted data using the regression model are to the true measured value of the spirometer, based on the sum of squared errors [24,25]. It is given by:

(1) $$R^{2}=1-\frac{SS_{res}}{SS_{total}}$$

where *SS_total_* and *SS_res_* are given by:

(2) $$SS_{total}=\overset{n}{\underset{i=1}{\sum }}(y_{i}-\bar{y})$$

(3) $$SS_{res}=\overset{n}{\underset{i=1}{\sum }}(y_{i}-{\hat{y}}_{i})$$

where *y_i_* is the volume measured using the spirometer (the ground truth), $\bar{y}$ is the average value of the measured values, ${\hat{y}}_{i}$ is the predicted regression model volume, and *n* is the number of signals tested (sample size).

The *MSE* is a measure of the average error between the measured and the predicted values and is given by:

(4) $$MSE=\frac{1}{n}\overset{n}{\underset{i=1}{\sum }}(y_{i}-{\hat{y}}_{i})$$

The *RMSE* is the square root of mean square error and is given by:

(5) $$RMSE=\sqrt{\frac{1}{n}\overset{n}{\underset{i=1}{\sum }}(y_{i}-{\hat{y}}_{i})}$$

The *MAE* is a measure of the absolute error between the measured and the predicted volumes. It is given by:

(6) $$MAE=\frac{1}{n}\overset{n}{\underset{i=1}{\sum }}\left|y_{i}-{\hat{y}}_{i}\right|$$

## 3. Results

**Figure 6:**
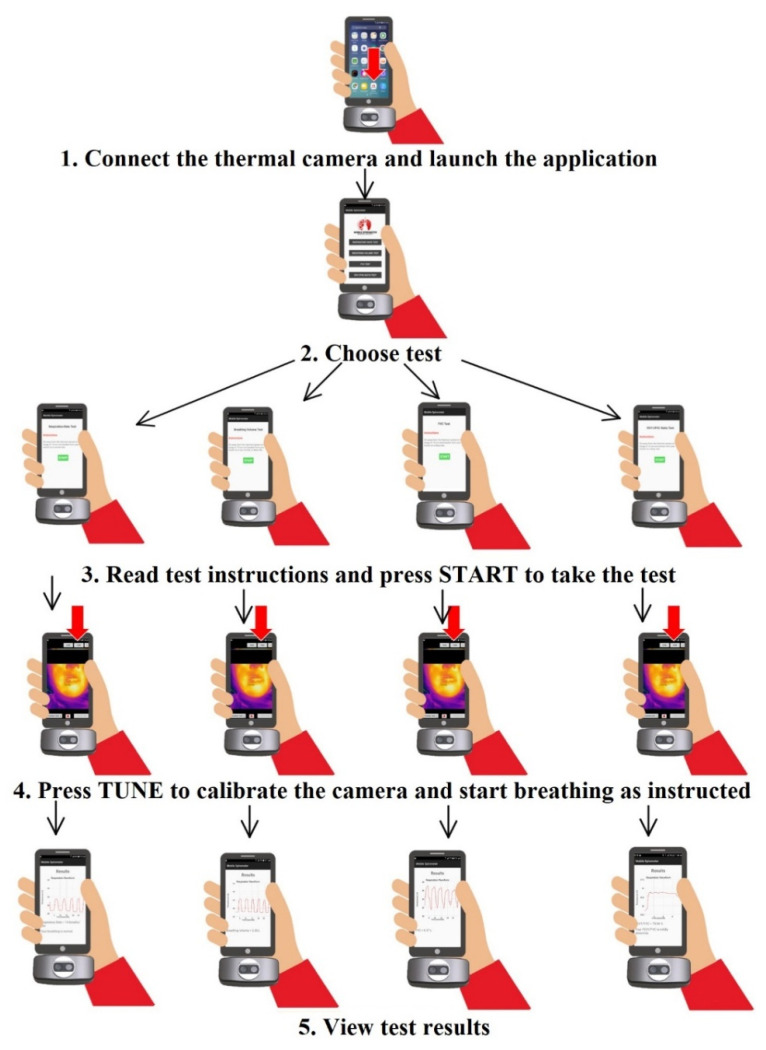
Mobile application functionality of the proposed system.

The complete proposed system was implemented using a mobile application with the functionality illustrated in Figure 6. The system can provide two types of measurements: the first one is the respiration rate and the second one is the respiration volume measurement, which depends on the measurement protocol. Three volume measurements were programmed in the app: the normal respiration volume (tidal volume), the functional reserve capacity (FVC) or deep breathing, and the FEV1/FVC ratio.

### 3.1. Respiration Signal and Respiration Rate Measurements

**Figure 7:**
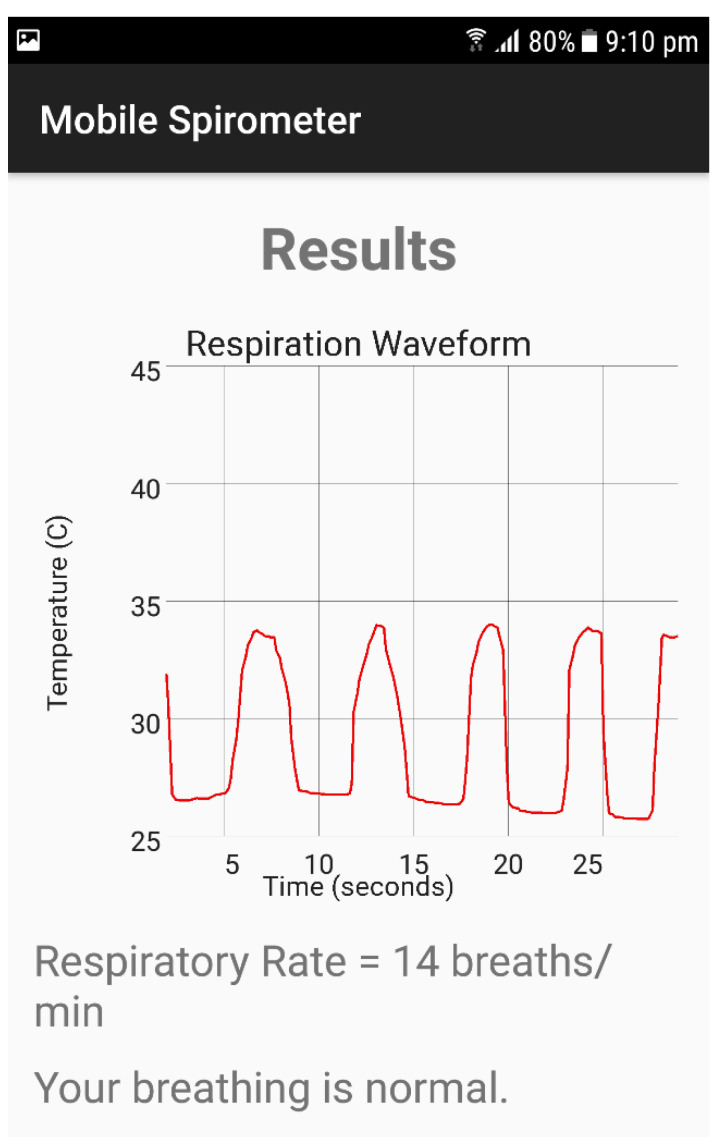
The interface of the mobile application for respiration rate measurements.

This module was implemented as a mobile app with a workflow described in Figure 2. The application was programmed using Java programming language in the environment of the Android studio. Thermal image acquisition was performed using the mobile thermal camera software development kit, and facial recognition and the area of interest were implemented using the OpenCV image processing library. Figure 7 shows the interface of the mobile application with the respiration rate measurement and the respiration signal. The two measurements were recorded at a distance of around 20 cm from the subject’s face with a duration of 20 s as a default value. The recording was carried out at room temperature (23 °C).

**Figure 8:**
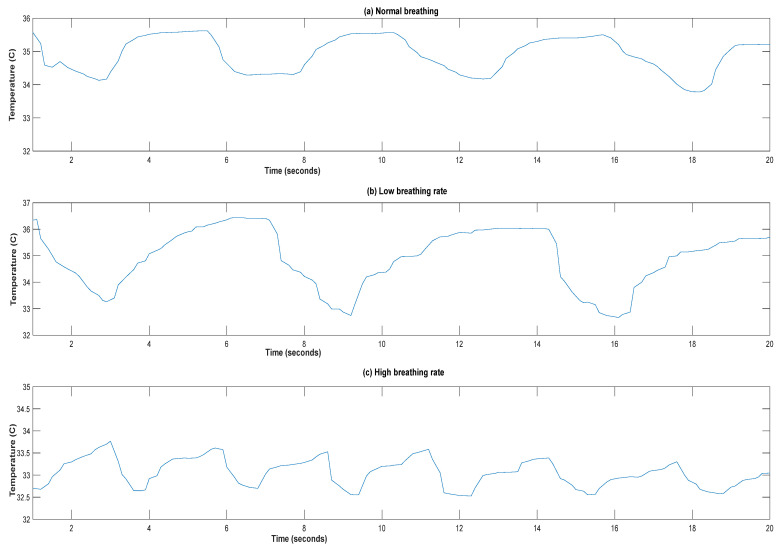
Respiration signal recorded at different rates: (**a**) normal breathing rate, (**b**) low breathing rate, and (**c**) high breathing rate.

The recorded respiration signal was exported to a back-end server for further measurements. Figure 8 shows an example of these recordings at three different respiration rates.

**Figure 9:**
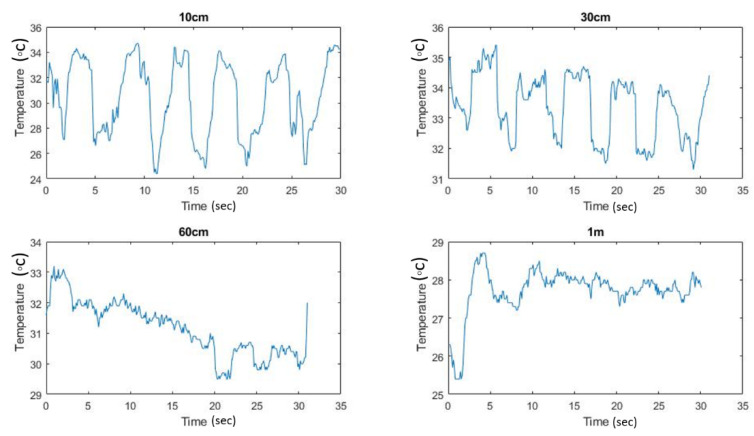
Respiration signals recorded at 10 cm, 30 cm, 60 cm, and 100 cm.

To explore the effect of the camera distance from the subject’s head, four respiration signals were recorded at 10 cm, 30 cm, 60 cm, and 100 cm, as shown in Figure 9.

### 3.2. Spirometry Measurement System

The spirometry measurement system was completely performed in the Python 3.7 programming environment. The package Scikit-learn 0.24.1 was used for this purpose [25]. The four regression models mentioned earlier were considered. The regression testing and training were carried out using a 10-fold cross-validation technique. The performance of the regression of four techniques is listed in Table 1. The performance measures indicated that the decision trees, along with the gradient boosting regression methods, had better performance in comparison with the other two methods, with slightly superior performance for the decision trees.

**Figure 10:**
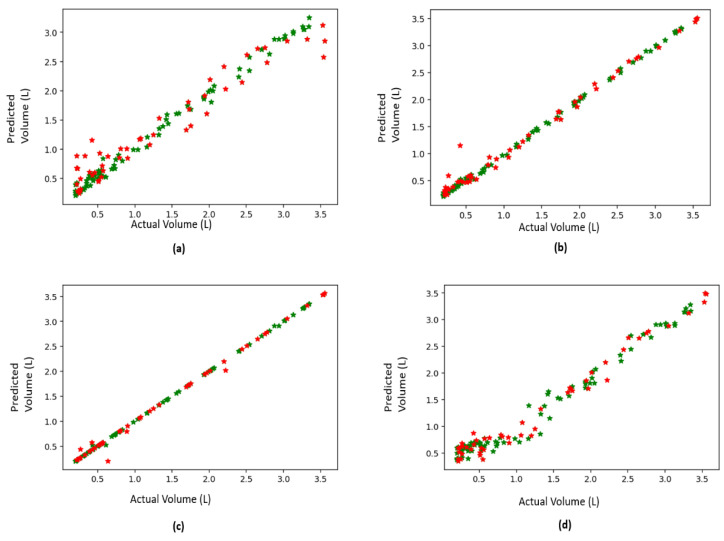
Results of the 10-fold cross-validation testing procedure: (**a**) random forests regressor, (**b**) gradient boost regressor, (**c**) decision trees regressor, and (**d**) adaptive boosting regressor.

Testing of the proposed methods was carried out using a 10-fold cross-validation procedure. Figure 10 shows the agreement between the predicted change in volume of the method in comparison with the measured spirometer value. A perfect agreement is indicated by a line of a slope of 1. The performance of the decision trees method is evident in Figure 8c and the performance measures in Table 1.

### 3.3. Spirometry System Testing

**Figure 11:**
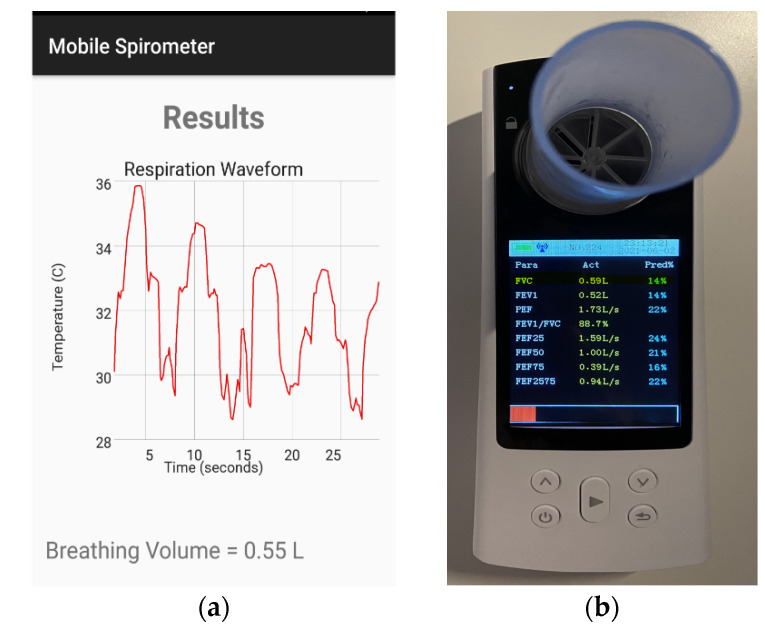
Measured normal breathing volume (tidal volume) (**a**) using the mobile application with a thermal camera and (**b**) using the conventional spirometer.

**Figure 12:**
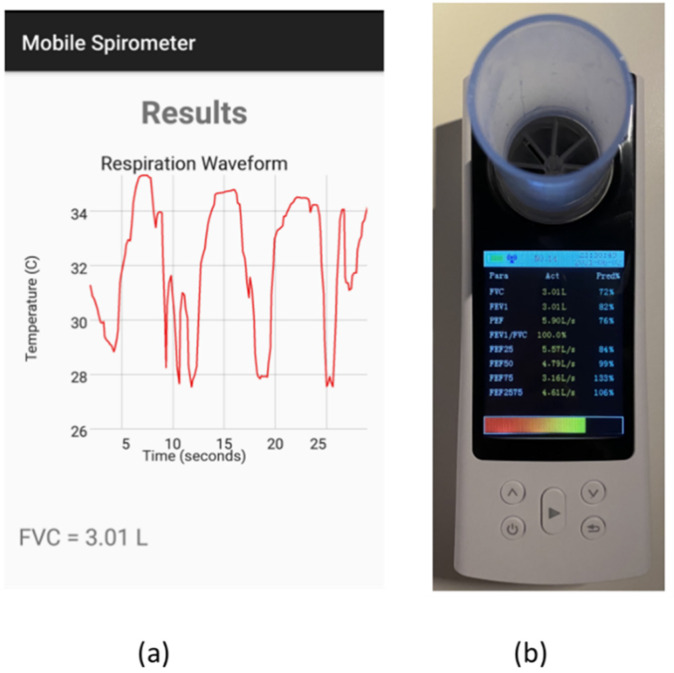
FVC test measurement: (**a**) mobile app reading; (**b**) conventional spirometer reading.

The complete proposed system was tested for volume measurements with several breathing volumes and compared with the reading provided by the conventional spirometer. The first test was performed on a normal subject with a normal breathing rhythm. As shown in Figure 11, a breathing volume of 0.55 L was recorded using the mobile spirometer application, while the conventional spirometer recorded a breathing volume of 0.59 L. Another breathing test was performed to measure the FVC of a normal subject. The proposed system showed a measured volume of 3.01 L, which was exactly the same volume measured by the conventional spirometer. The measurements are shown in Figure 12.

**Figure 13:**
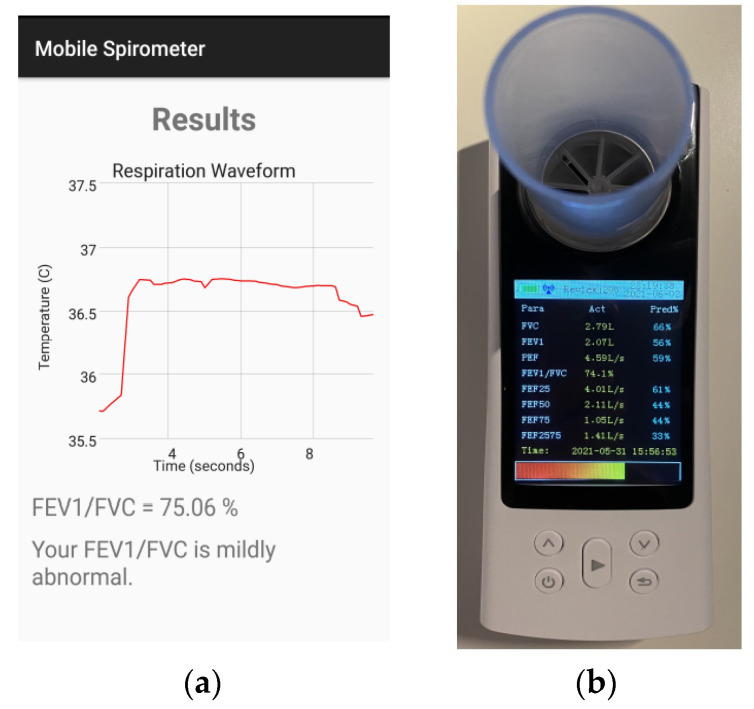
FEV1/FVC measurement (**a**) using the mobile app and (**b**) using the Contec spirometer.

The last measurement test was the FEV1/FVC ratio. The FVC was measured as mentioned previously, while the FEV1 was measured based on the change in respiration volume with time (1 s). Figure 13 shows the FEV1/FVC measurement using both the mobile app and the Contec spirometer.

## 4. Discussion

This work presents a feasibility study for respiratory measurements using a mobile thermal camera. The measurements were made by a first-generation FLIR I mobile thermal camera. Although there are more advanced thermal cameras now, due to the need for temperature monitoring because of the COVID-19 pandemic, the camera provided good results for respiration rate measurements at a close distance. The FLIR I is a very basic first-generation mobile thermal camera with low resolution compared with other advanced cameras. The camera provides the highest possible accuracy by automatically calibrating itself according to the scene temperature by calculating the emissivity of the objects, the reflected temperature, and the distance; it then returns the actual temperature of the object. The accuracy of the recorded temperature was reported in the literature to be around 3 °C [26]. The effect of the measurement accuracy was minimized in this study for two reasons: first, we measured the average temperature of the area of interest and not pixel by pixel temperature, which resulted in reducing errors in the measured temperature. The second reason is that the measured signal represents the change in temperature and not the actual temperature, which cancels the effect of errors that may appear during the calibration of the camera.

In the current study, the distance was around 20 cm; greater distances resulted in degradation of the signal quality, as shown in Figure 6. Therefore, higher-quality cameras may result in a better respiration signal and thus better measurements at higher distances. One of the most promising applications of respiratory rate measurements is sleep apnea (cessation of breathing). The mobile thermal camera, along with the mobile application, could provide a non-contact and hassle-free system to detect apnea during sleep, as the current clinical apnea monitoring system requires wiring to the patient’s body.

The FLIR I thermal camera has an image acquisition rate of nine frames per second; therefore, the average temperature has a sampling rate of 9 Hz. The respiration rate varies with age and the maximum breathing rate can reach up to 55 breaths per minutes in neonates [27]. This means that the maximum breathing frequency is 1.1 Hz and therefore, the current image acquisition rate of the FLIR I is enough to provide a faithful representation of the respiration waveform. At the same time, higher imaging rates would provide a better representation of the respiration signal and hence better performance.

The change in the lung volume (spirometry) measurement was performed using an AI approach based on different regression models. The decision trees regressor showed excellent performance in predicting the change in volume due to respiration, with a mean R-square value of 0.9998 and a mean square error of 0.0023, as listed in Table 1. Moreover, the AI system was built (training and testing) using a range of volumes (0.2 L to 3.5 L), which makes it appropriate for a wide range of subjects and ages. Several studies have adopted a non-contact approach for respiration measurements [8,28,29]. Brieva et al. [30] estimated the respiratory rate using a Hermite magnification technique and a convolutional neural network (CNN). Their contactless system monitored the subjects’ chest movements with and without an ROI (region of interest), and they evaluated the performance of their methods using the mean average error. With and without an ROI, the respiratory rate was estimated with a mean average error of 1.83 ± 1.61% and 3.28 ± 3.33%, respectively. Liu et al. [31] proposed an imaging-based approach to determine spirometry parameters such as FEV1, FVC, and PEF. They captured images of the subjects’ faces and shoulders during inspiration and expiration using a webcam. Their method showed fair performance, with a root mean square error of 0.27, 0.18, and 0.56 and an average error of 8.5%, 6.9%, and 7.7% for FEV1, FVC, and PEF, respectively. However, using infrared thermography, Pereira et al. [32] estimated the breathing rate with a mean absolute error of 0.33, 0.55, and 0.96 bpm (breaths per minute). One of the most recent studies is the study by Schoun et al. [28]. They used an in-vitro benchtop thermal imaging setup to acquire simulated measurements. They used a recurrent network model (AI) to predict the respiration signal, and from the predicted signal, they made respiration measurements. Using RMSE, their LSTM neural network predicted the expiratory volume from human tests with an average error of 10.61%. However, in this study, the change in volume due to respiration was predicted directly using AI regression models.

## 5. Conclusions and Future Work

The current study presents a successful approach for respiratory measurements of the respiration rate and volume change. It could provide a means for a non-contact, continuous, and accurate method for the monitoring of chronic and acute pulmonary diseases. However, more steps forward are still needed to improve the quality of the thermal signal measurements by using a more advanced thermal camera and probably more advanced image processing techniques for detection of the area of interest in the face. Advanced thermal cameras can provide better signal quality at longer distances and, at the same time, better image quality. Moreover, an increase in the image acquisition rate should significantly improve the proposed system. This study could also be further developed to be used for simultaneous multiple subject monitoring and measurements, especially with the current COVID-19 pandemic. This study could also be tested in a clinical setup with diseases such as asthma, chronic obstructive pulmonary disease (COPD), and pneumonia. Moreover, this study could be performed against different temperature background temperatures to check the reliability of the measurements in a neutral thermal zone. This will require a special setup that can produce different thermal background temperatures.

## Figures and Tables

**Table 1:** Summary of the regression methods used.

Performance Measure/ Regression Method	Random Forest	Gradient Boosting	Adaptive Boosting	Decision Trees
R-square	0.9409	0.9948	0.9558	0.9998
Mean absolute error	0.1886	0.0532	0.1724	0.0023
Mean squared error	0.0670	0.0049	0.0423	0.0002
Root mean squared error	0.2484	0.0687	0.2054	0.0088
